# Integrated Multi-Omics Analysis Model to Identify Biomarkers Associated With Prognosis of Breast Cancer

**DOI:** 10.3389/fonc.2022.899900

**Published:** 2022-06-10

**Authors:** Yeye Fan, Chunyu Kao, Fu Yang, Fei Wang, Gengshen Yin, Yongjiu Wang, Yong He, Jiadong Ji, Liyuan Liu

**Affiliations:** ^1^ School of Mathematics, Shandong University, Jinan, China; ^2^ Zhongtai Securities Institute for Financial Studies, Shandong University, Jinan, China; ^3^ Department of Breast Surgery, The Second Hospital, Cheeloo College of Medicine, Shandong University, Jinan, China; ^4^ Institute of Translational Medicine of Breast Disease Prevention and Treatment, Shandong University, Jinan, China

**Keywords:** multi-omics, survival prediction, differential network, breast cancer, prognosis

## Abstract

**Background:**

With the rapid development and wide application of high-throughput sequencing technology, biomedical research has entered the era of large-scale omics data. We aim to identify genes associated with breast cancer prognosis by integrating multi-omics data.

**Method:**

Gene-gene interactions were taken into account, and we applied two differential network methods JDINAC and LGCDG to identify differential genes. The patients were divided into case and control groups according to their survival time. The TCGA and METABRIC database were used as the training and validation set respectively.

**Result:**

In the TCGA dataset, C11orf1, OLA1, RPL31, SPDL1 and IL33 were identified to be associated with prognosis of breast cancer. In the METABRIC database, ZNF273, ZBTB37, TRIM52, TSGA10, ZNF727, TRAF2, TSPAN17, USP28 and ZNF519 were identified as hub genes. In addition, RPL31, TMEM163 and ZNF273 were screened out in both datasets. GO enrichment analysis shows that most of these hub genes were involved in zinc ion binding.

**Conclusion:**

In this study, a total of 15 hub genes associated with long-term survival of breast cancer were identified, which can promote understanding of the molecular mechanism of breast cancer and provide new insight into clinical research and treatment.

## Introduction

Gene variation and expression play an important role in the development of cancer. Breast cancer ranks as the greatest killer among women’s cancers (1). Therefore, research on genes related to long-term survival in breast cancer is of great significance for medical workers, in order to enable the development of targeted drugs and formulation of reasonable plans.

The development of high-throughput sequencing technology provides a unique opportunity for the prognostic prediction of breast cancer (2). Most of the early breast cancer studies were conducted based on single omics data such as gene expression (3, 4). For example, 70 genes related to the survival of breast cancer patients were identified by feature screening in 295 samples of breast cancer gene expression data using multivariate analysis (5). However, the development of cancer is a multiplex, multi-factorial process, involving a variety of molecular-level biological mechanisms; it is difficult for single omics analysis to elucidate the biological process of breast cancer development (6). The integration of multiple omics data is conducive to comprehending the mechanism of disease occurrence and development and can inject new blood into biological research (7). Many studies have found that integrating multiple omics data can improve clinical classification performance (8–10). Zeng integrated radiological and genomic data to predict the survival of clear-cell renal carcinoma using multiple machine learning classifiers such as logistic regression and support vector machine methods, and found that multi-omics models were more accurate than single-omics models (11).

The occurrence and development of cancer are often related to interactions between multiple genes. The heterogeneity of genomic data and the characteristics of interaction analysis result in limitations for traditional statistical methods in the application of the whole genome (12). Differential network estimation has become an important tool for exploring biological mechanisms, and the interaction patterns can provide opportunities for screening important biomarkers in disease research, which has a wide range of biological and clinical research significance (13–15). Kim used a graph-based data-fusion approach to treat multiple omics data as different nodes in a heterogeneous network for the clinical postoperative prediction of the stage, grade, and survival of ovarian cancer patients (16). Gatto constructed genome-scale metabolic-network models for 13 cancers based on the cancer genome atlas (TCGA) dataset and found that different cancers showed similar metabolic networks (17). However, most differential network models are based on single omics data, and few studies have combined multiple omics data for differential network analysis.

In this study, two advanced differential network methods for continuous and discrete data were combined to identify the differential genes and interaction networks related to breast cancer. Gene expression profiles, somatic mutations, and copy number variations (CNVs) were collected from the cancer genome atlas (TCGA) and molecular taxonomy of breast cancer international consortium (METABRIC). By integrating genomic and transcriptomic data, we screened prognostic markers and constructed gene-interaction networks related to the long-term survival of breast cancer patients. Functional enrichment analysis was used to identify the important biological processes associated with breast cancer. The current study provides insights into the molecular mechanisms underlying breast cancer prognosis and will support the development of clinical trials and breast cancer research.

## Materials and Methods

### Data Sources and Preprocessing

Omics data for mRNA gene expression, CNVs, and mutations were integrated into our study. The TCGA database was used as the training set (18). The gene expression profiles with the HTSeq‐FPKM format of BRCA samples and mutation profiles were obtained directly from the data portal of TCGA (https://portal.gdc.cancer.gov/). We used R to convert RNAseq data from fragments per kilobase million (FPKM) format to transcripts per million (TPM) format. CNV profiles and survival data were downloaded from http://xena.ucsc.edu/, and the validation set METABRIC database was downloaded from http://www.cbioportal.org/ (19–22).

**Figure 1:**
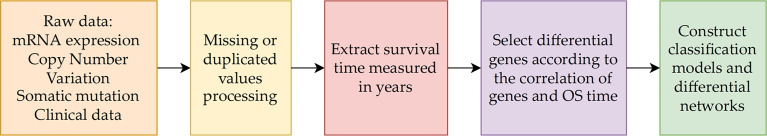
The workflow of our study.

The specific steps of our study were as follows: (1) The original data were obtained. (2) Gene expressions with ≥ 5% missing values were deleted, and those with < 5% missing values were interpolated with the median. The expression values of genes with repeated sample IDs were replaced by the mean values. (3) The survival time (“OS.time” in TCGA and “OS_MONTHS” in META) was extracted, and the units were uniformly converted into years. (4) The coefficients of the distance correlations between genes and the survival time were calculated. (5) We selected the common differential genes in three omics of the TCGA dataset to train the classification model and construct the gene-interaction network. Finally, 966 patients in TCGA and 1,866 patients in METABRIC were used in our study. The workflow of our study is shown in **Figure 1**.

We considered four survival time categories: 1-year, 3-year, 5-year, and 10-year. The classification performance was measured using the receiver operating characteristic (ROC) curve, Kaplan-Meier curve, area under the ROC curve (AUC), and accuracy. The high-risk group and low-risk group in Kaplan-Meier curves were truncated by the cutoff calculated by ROC curves. The differential networks were drawn to identify genes associated with breast cancer prognosis.

### DC-SIS Variable Selection

The dimension of biological data is too large, and they contain many genes with little significance. To effectively utilize the data and reduce the cost of machine learning, variable selection is required. We used the sure independence screening procedure based on distance correlation (DC-SIS) method to select differential genes using the “energy” package (v1.7-8) in R (23).

The DC-SIS method measures the correlation between two random vectors according to their distance correlation coefficients (24). The distance covariance of two random vectors *u* and *v* is defined as


$$\text{dcov}\left(u,v\right)=\int_{R^{d_{u}+d_{v}}}{∥\varphi_{u,v}\left(t,s\right)-\varphi_{u}\left(t\right)\varphi_{v}\left(s\right)∥}^{2}\omega \left(t,s\right)\text{d}t\text{d}s,$$

in which *d_u_* and *d_v_* are the dimensions of *u* and *v*, respectively; *φ_u_* (*t*) and *φ_v_* (*s*) are their respective eigenfunctions; *φ_u,v_* (*t*,*s*) is their joint eigenfunction; and


$$\begin{array}{l} \omega \left(t,s\right)={\left\{c_{d_{u}}c_{d_{v}}{∥t∥}_{d_{u}}^{1+d_{u}}{∥s∥}_{d_{v}}^{1+d_{v}}\right\}}^{-1},\;c_{d}=\pi^{\left(1+d\right)/2}/\text{Γ}\left\{\left(1+d\right)/2\right\}. \end{array}$$

The distance correlation of *u* and *v* is obtained by dividing their distance covariance by the product of their distance standard deviations, which is


$$\text{dcor}\left(u,v\right)=\frac{\text{dcov}\left(u,v\right)}{\sqrt{\text{dcov}\left(u,u\right)\text{dcov}\left(v,v\right)}}.$$

### Classification and Differential Network Analysis

**Figure 2:**
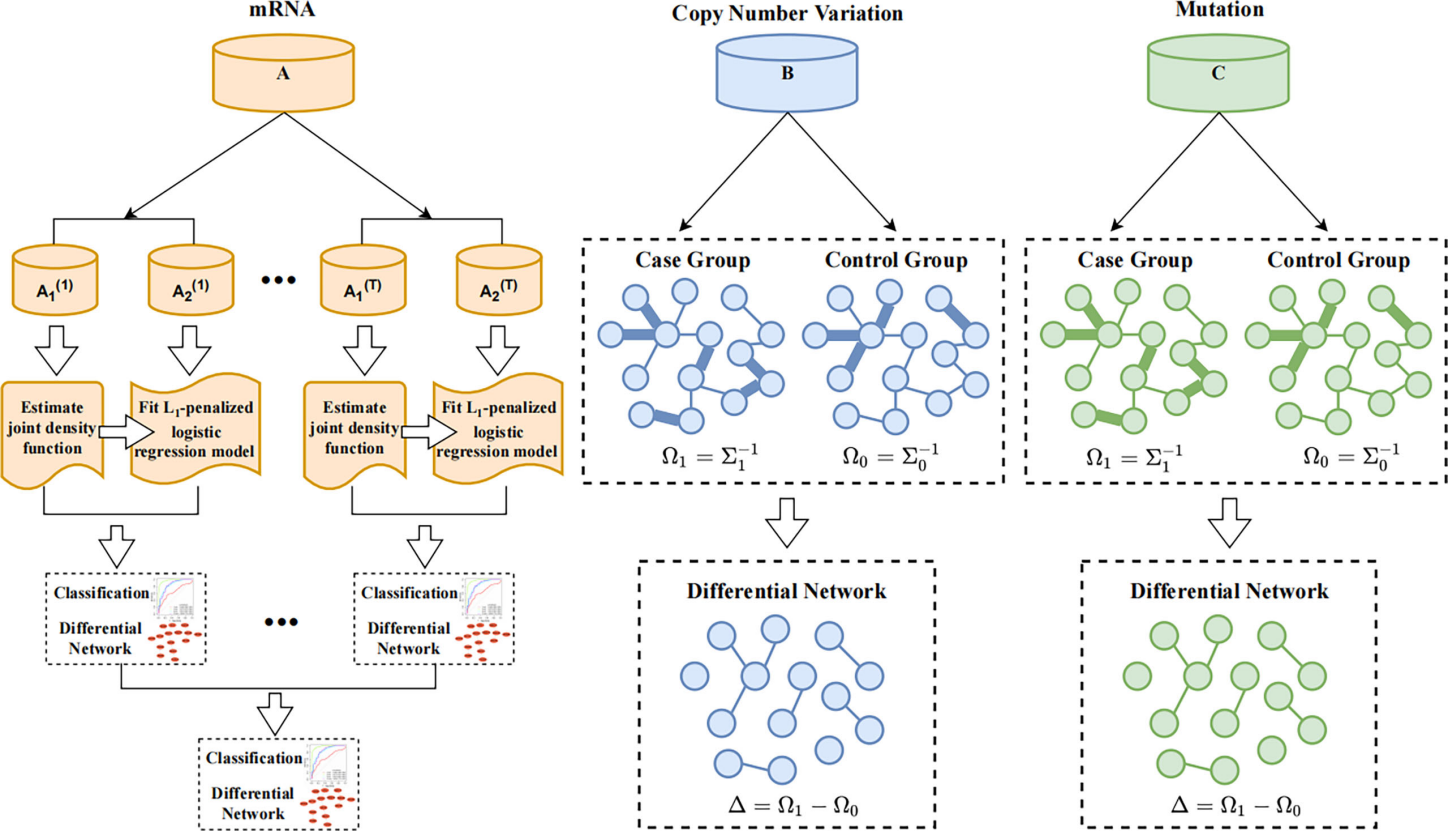
Flow chart of JDINAC and LGCDG method. **A–C** represent mRNA expression, copy number variation, and somatic mutation datasets.

Since gene expression data are continuous variables, whereas CNVs and mutations are discrete variables, we applied two advanced differential network estimation methods to infer the interaction networks and differential genes. In this paper, the differential network consists of difference edges that filtered out from at least two omics of data. We used Cytoscape (25) to plot the differential network. A flow chart of the study is shown in **Figure 2**. Among them, we used TCGA as the training set and METABRIC as the verification set. The omics data mRNA, CNV and somatic mutation in TCGA and mRNA and CNV in METABRIC were used to construct the differential network respectively.

For continuous-variable mRNA data, we referred to the joint density-based non-parametric differential interaction network analysis and classification (JDINAC) method to measure the interaction between the two variables, and then used L_1_-Penalized logistic regression to build the prediction model and screen the differential genes (26). A total of 50% of the data were used to fit the joint density function, and 50% were used to fit the regression model; the number of data splits was 100. The mean of the predicted values is taken as the final prediction probability.

JDINAC is a nonparametric kernel method that considers gene-gene interaction, which is characterized by estimating the conditional joint density of gene pairs (26). If (*x_i_, x_j_*) denotes one gene pair, the response variable is *y* = {0,1}; patients with short survival time were labeled 1, and those with long survival time were labeled 0. For example, in the case of 1-year classification, samples with survival time less than or equal to 1 year were labeled 1, and those with survival time greater than 1 year were labeled 0. *f_ij_* (*x_i_,x_j_*) and *g_ij_* (*x_i_,x_j_*) represent the class conditional densities for class 1 and class 0, respectively, where *f_ij_* (*x_i_,x_j_*) = *P*((*x_i_,x_j_*) | *y* = 1) and *g_ij_* (*x_i_,x_j_*) = *P*((*x_i_,x_j_*) | *y* = 0). The log ratio of the two-dimensional class conditional density $ln\frac{f_{ij}\left(x_{i},x_{j}\right)}{g_{ij}\left(x_{i},x_{j}\right)}$ was used as the classification predictor variable $ln\frac{f_{ij}\left(x_{i},x_{j}\right)}{g_{ij}\left(x_{i},x_{j}\right)}>0$ indicates that the gene pair is more closely related in class 1, whereas $ln\frac{f_{ij}\left(x_{i},x_{j}\right)}{g_{ij}\left(x_{i},x_{j}\right)}$ indicates that there is stronger dependency between genes in class 0. Based on the *L*_1_-penalized logistic regression model, the prediction accuracy is improved in the multivariate classifier, and the logistic model can be


$$logit\left(P\right)=\beta_{0}+\sum_{i=1}^{p}\sum_{j=i+1}^{p}\beta_{ij}ln\frac{f_{ij}\left(x_{i},x_{j}\right)}{g_{ij}\left(x_{i},x_{j}\right)},\sum_{i=1}^{p}\sum_{j=i+1}^{p}\left|\beta_{ij}\right|<c,\;c>0.$$

To explore the differential networks of the discrete-variable CNV and mutation data, we applied the latent Gaussian copula differential graphical (LGCDG) model, which defines the differential network as the difference between the precision matrices of the short-term (labeled 1) and long-term (labeled 0) survival groups (27). We transferred the CNV data into binary variables; specifically, the non-zero elements were encoded as 1, indicating that the copy number is out of the normal range.

LGCDG assumes that the 0/1 binary data ***D*** = (*D*_1_, *D*_2_, … , *D_p_*)^T^ ∈ {0,1}*^p^* satisfies the latent Gaussian copula model (LGCM), that is, the binary data are generated by discretizing a latent continuous variable at some unknown cutoff. In this assumption, the continuous variable follows a non-paranormal distribution, which is *X* ~ NPN(**0**,**Σ**,*f*), and the binary variable can be *D_j_* = *I*(*X_j_* > *C_j_*); then, *D* ~ LGCM(**Σ**,Λ), where Λ*_j_* = *f_j_* (*C_j_*).

We assume *D* ^1^ ~ LGCM(**Σ** ^1^,Λ^1^) and *D* ^0^ ~ LGCM(***Σ*** ^0^,*Λ* ^0^) are the binary data from the case and control group, respectively. The differential network is defined as the difference between the two precision matrices, denoted by **Δ** = (**Σ** ^1^)^-1^ – (**Σ** ^0^)^-1^. The estimator of **Δ** can be obtained by solving the following optimization problem:


$$\arg min{\left|\Delta \right|}_{1},\;\mathrm{subject}\;\mathrm{to}\;{\left|\left({\hat{b}}^{1}⊗{\hat{b}}^{0}\right)\text{Vec}\left(\Delta \right)-\text{Vec}\left({\hat{b}}^{1}-{\hat{b}}^{0}\right)\right|}_{\infty }\leq \lambda_{n},$$

where ${\hat{b}}^{1}$ and ${\hat{b}}^{0}$ are the Kendall’s tau rank-based correlation matrix estimators for **Σ** ^1^ and **Σ** ^0^.

The underlying differential network of binary data can be inferred through the LGCDG method, which provides a deeper understanding of the unknown mechanism than that among the observed binary variables.

### GO Function Enrichment

Gene ontology (GO) enrichment analysis was performed to better understand the biological functions of the differential genes selected by DC-SIS method, and the “clusterProfiler” package (v4.2.1) and “org.hs.eg.db” package (v3.14.0) in R were used (28). With reference to the whole human genome, significant functional categories and the biological functions of the differential genes were identified.

## Results

### DC-SIS Variable Selection

The coefficients of the distance correlations between genes and survival time were calculated; a total of 140 common genes for three omics were screened (listed in **Table S1**). The differential genes were considered as the genes highly expressed and mutated in breast cancer and were used in the following study.

### Classification and Differential Network in TCGA

**Figure 3:**
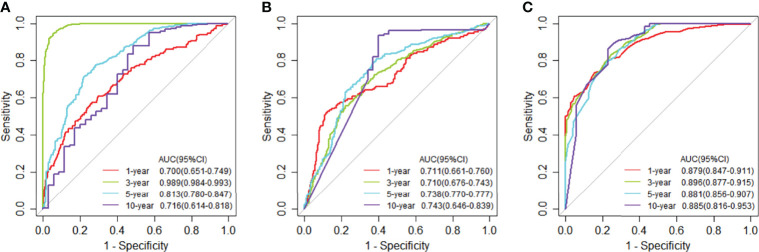
Time-dependent receiver operating characteristic (ROC) curves at 1, 3, 5, and 10 years of mRNA expression data in TCGA. **(A)** The ROC curves for JDINAC classifier. **(B)** The ROC curves for logistic regression classifier. **(C)** The ROC curves for random forest classifier.

**Figure 4:**
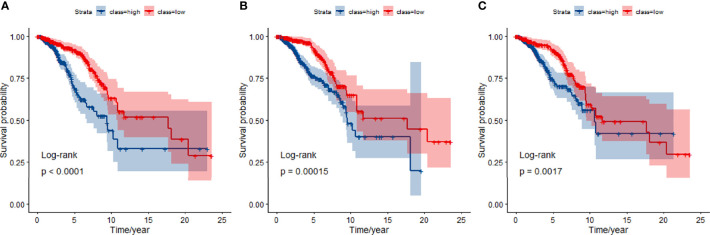
Kaplan-Meier curves for overall survival at 5-year classifiers of mRNA expression data in TCGA. **(A)** Kaplan-Meier curves for JDINAC classifier, **(B)** Kaplan-Meier curves for logistic regression classifier, and **(C)** Kaplan-Meier curves for random forest classifier.

We compared the JDINAC model with classical binary classifiers logistic regression and random forest using 5-fold cross-validation; the ROC curves and 5-year Kaplan-Meier curves of mRNA gene expression data are shown in **Figures 3**, **4**. The other Kaplan-Meier curves in TCGA dataset were shown in **Figure S1**. The classification performance of the three classifiers was measured in terms of the AUC, specificity, sensitivity, and accuracy (**Table S2**). The results show that our model has better classification performance than logistic regression model, and it can achieve comparable performance to the random forest method. In addition, the AUCs of JDINAC are all above 0.7 and even reached 0.989 in the 3-year classification category, which is sufficient to prove the efficient classification performance of JDINAC. The Kaplan-Meier curves also show that our method has better classification ability than the other two models.

**Figure 5:**
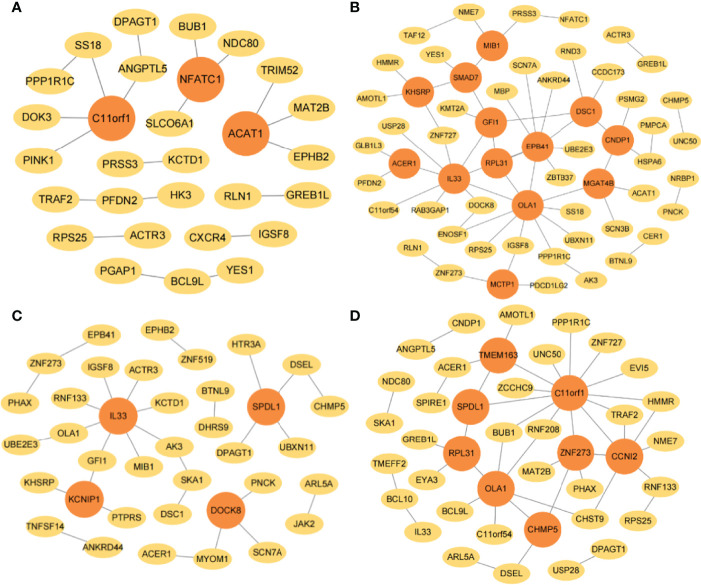
The gene–gene interaction network in TCGA. The selected interaction networks for **(A)** 1-year, **(B)** 3-year, **(C)** 5-year, and **(D)** 10-year categories. The orange circular nodes represent hub genes.

The interaction networks of genes were performed by combining JDINAC and LGCDG, in which the three omics data were integrated. The differential network is composed of the common edges screened out from omics data. Genes are represented by nodes, the interactions between genes are represented by edges between nodes, and genes with at least three edges are regarded as hub genes. The hub genes were identified under four taxonomic conditions, of which C11orf1, OLA1, RPL31, SPDL1, and IL33 were identified in at least two interaction networks, and all of these genes were found in 5- or 10-year interaction networks (**Figure 5**).

### Classification and Differential Network in METABRIC

**Figure 6:**
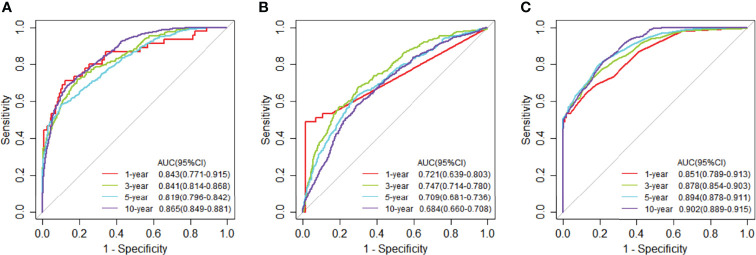
Time-dependent receiver operating characteristic (ROC) curves for 1-, 3-, 5-, and 10-year mRNA expression data in METABRIC. **(A)** The ROC curves for JDINAC classifier. **(B)** The ROC curves for logistic regression classifier. **(C)** The ROC curves for random forest classifier.

**Figure 7:**
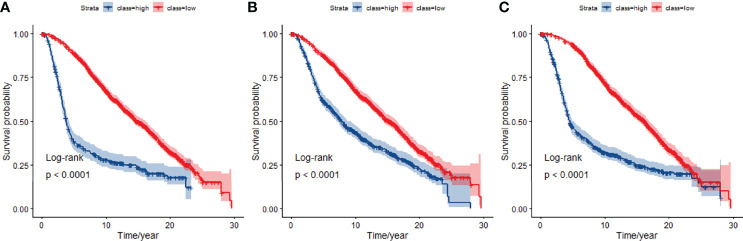
Kaplan-Meier curves for overall survival at 5-year classifiers of mRNA expression data in METABRIC. **(A)** Kaplan-Meier curves for JDINAC classifier, **(B)** Kaplan-Meier curves for logistic regression classifier, and **(C)** Kaplan-Meier curves for random forest classifier.

We used the selected 140 differential genes in TCGA to evaluate the performance in METABRIC. Mutation data were not included in the model due to insufficient sample size. The JDINAC classification performance of the mRNA expression data was compared with the logistic regression and random forest methods using 5-fold cross-validation, and the ROC and Kaplan-Meier curves are shown in **Figures 6**, **7**. The other Kaplan-Meier curves in METABRIC dataset were shown in **Figure S2**. The AUCs, specificities, sensitivities, and accuracies of the three classifiers are listed in **Table S3**. The classification performance of JDINAC is as good as that of random forest method, and is better than that of logistic regression. The AUCs of JDINAC were all above 0.8, which indicates that the JDINAC method showed excellent classification performance.

**Figure 8:**
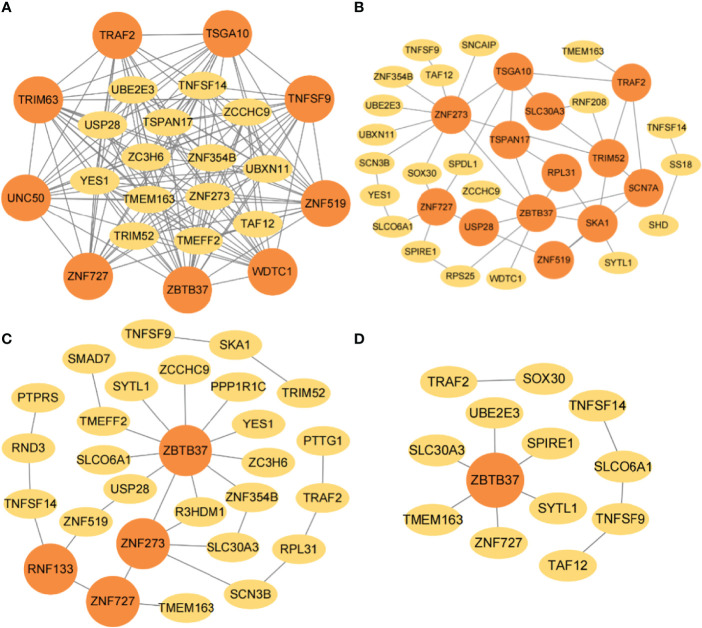
The gene-gene interaction network in METABRIC. The selected interaction networks for **(A)** 1-year, **(B)** 3-year, **(C)** 5-year, and **(D)** 10-year categories. The orange circular nodes represent hub genes.

The gene-interaction networks for four classification categories were determined by combining JDINAC and LGCDG methods, and the identified hub genes are marked by orange circles (**Figure 8**). The results show that ZNF273, ZBTB37, TRIM52, TSGA10, ZNF727, TRAF2, TSPAN17, USP28, and ZNF519 were identified in at least two interaction networks, in which ZNF273, ZBTB37, and ZNF727 are related to 5-year or 10-year survival in breast cancer. Additionally, it is interesting that RPL31, TMEM163, and ZNF273 were selected as hub genes in both the TCGA and METABRIC databases, this is a finding that cannot be ignored.

### GO Function Enrichment

**Figure 9:**
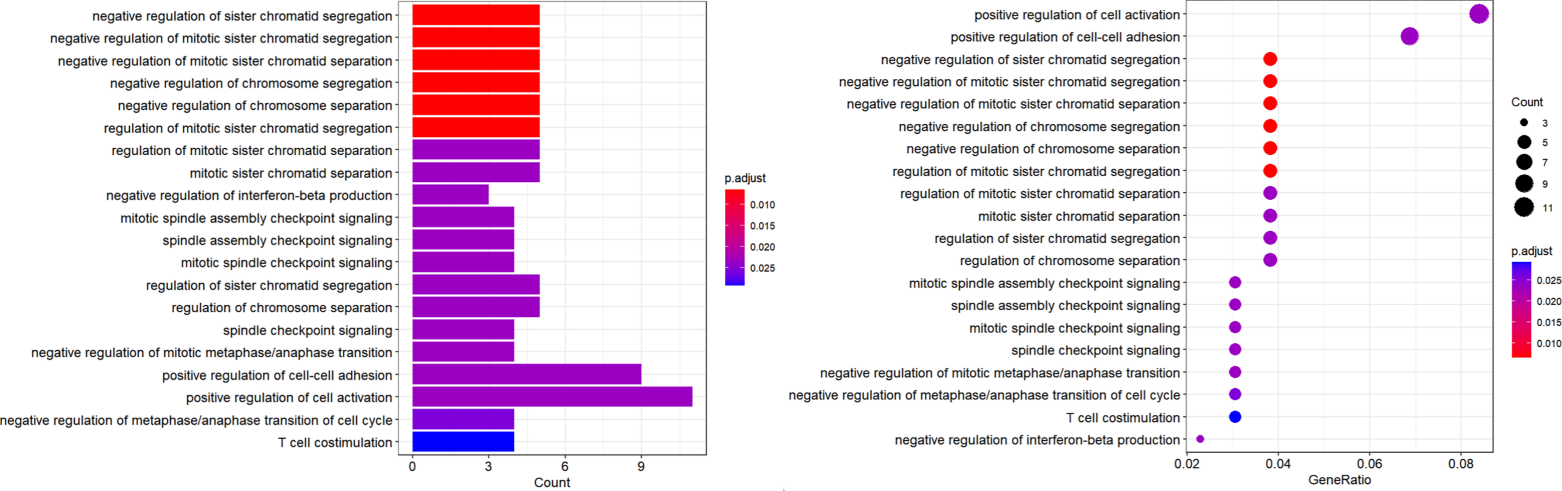
GO functional enrichment of the differential genes.

We performed GO enrichment analysis to assess which functional categories of genes were most connected to the prognosis of breast cancer. Enrichment analysis revealed that these differential genes were significantly enriched in 34 GO terms, mainly associated with regulation of cell-cell adhesion, positive regulation of cell activation, and positive regulation of leukocyte activation (**Figure 9**). Combining these results with TCGA and METABRIC data, a total of 15 genes related to the prognosis of breast cancer were screened out. The GO terms enriched by the 15 genes show that the metastasis and prognosis of breast cancer are closely related to zinc-ion binding (**Table S4**), which means that genes related to zinc-ion binding have significant reference value in the study of breast cancer prognosis. Among them, ZBTB37, ZNF273, ZNF519, ZNF727 and IL33 are all involved in the biological process of transcription DNA-templated, which can be used in targeted gene therapy.

## Discussion

The incidence of breast cancer ranks the highest among malignant tumors in females (1). With the improvement of medical technology, the mortality rate for breast cancer has decreased significantly. However, drug resistance, recurrence, and metastasis remain poorly addressed, resulting in low long-term survival (29). To improve the efficacy of treatment for breast cancer patients, in-depth research on potential prognostic molecular markers related to long-term survival is of great significance. In this study, we utilized multi-omics data from TCGA and METABRIC to construct gene-gene interaction networks and identify differential genes, which can provide an important basis for the clinical diagnosis of and medical research on breast cancer.

In order to avoid information redundancy, the pre-screening of differential genes is essential. We usually screen differential genes by calculating the correlation of variables according to a certain principle, such as the p-value, Pearson’s correlation coefficient, and Kendall’s tau correlation coefficient. However, the omics data include continuous and discrete data, and the traditional screening criteria often assume that the variables obey certain distributions and tend to ignore the sample information. Compared with traditional statistical methods, DC-SIS can deal with multiple response variables, regardless of whether the response variables are continuous, discrete, or classified. It ensures that all important variables can be selected in a sufficient sample size. In addition, it does not make any model assumptions about responses and predictors, thus making model misrepresentation unlikely.

In recent years, many survival prediction models have been developed to identify prognostic biomarkers. Researchers usually use Kaplan–Meier and time-dependent ROC curves to measure predictive performance (30, 31). However, these studies are only based on the probabilities calculated using a single prediction model, which lacks discernibility in long-term survival. Zhou et al. used high-dimensional embedding and residual neural network method to extract hub genes by analyzing multi-omics data of breast cancer, but only analyzed the hub genes of each omics, lacking comprehensive consideration of multiple omics (32). In this study, we divided patients according to survival time, and constructed gene-interaction networks. Then, we focused on the differential genes associated with 5-year and 10-year survival, which makes more sense for the long-term survival of breast cancer patients.

Public sequencing platforms such as TCGA and GEO provide abundant omics data for biological researchers and facilitate molecular mechanism and clinical research. However, these datasets are highly heterogeneous, which poses significant challenges for existing approaches of data integration. There are many studies using multiple omics data and data-integration methods to analyze the survival of breast cancer patients. However, studies that combine gene interactions with multi omics are rare. Most of the studies on gene interaction focus on single omics, while the studies on multiple omics data often consider the impact of a single gene and ignore the gene interaction (33–36). We overcame the limitations of omics-data heterogeneity and applied interaction-network methods that are more suitable for multiple data types to identify hub genes.

In this study, we identified genes associated with breast cancer prognosis. Interestingly, most of the screened genes are involved in protein binding and zinc-ion binding. The results indicate that the tumorigenesis and development of breast cancer are closely related to zinc-ion binding, which is consistent with the findings in previous studies (37–39). Many studies have found that zinc is significantly correlated with the carcinogenesis of various tissues and cells in the body, and a change in the zinc content in the human body is closely related to the occurrence and development of tumors (40–42). In addition, zinc deficiency can cause immune dysfunction, which can enhance the inflammatory effects of interleukin, inhibit the effects of interleukin on lymphocytes, and promote apoptosis, angiogenesis, and metastasis. Zinc is often involved in gene expression, the maintenance of protein and nucleic acid structure, intracellular molecular transport, and immune functions performed by zinc-finger proteins (43–45). Studies have shown that zinc lipoprotein is involved in cancer-related biological processes, can inhibit the proliferation and invasion of cancer cells, and has a protective effect on the occurrence of prostate cancer (46–48).

There are endless studies about breast cancer prognosis, and our method has several advantages compared with other methods. Firstly, we used multiple omics data for gene expression, copy number variations, and somatic mutations, making full use of multiple levels of biological information to make the study more complete. Secondly, the interaction between genes was taken into account. The correlation between genes was incorporated into the predictive variables to preliminarily explore the biological mechanisms of complex diseases. Thirdly, we classified different survival periods, mainly focusing on the genes related to long-term survival. Genes associated with 5-year and 10-year survival were identified, and their biological functions were analyzed. Finally, we solved the problem of data heterogeneity. Appropriate differential network approaches were used to estimate gene differential networks and identify hub genes.

The results for the selected genes can provide potential targets for the clinical diagnosis of breast cancer. Although we identified potential candidate genes for breast cancer prognosis using bioinformatics approaches, some limitations of this study need to be noted. First, our sample lacked clinical follow-up information, and the database analysis based on publicly available data is not convincing enough; it needs to be verified by further clinical trials. We also lack the comprehensive consideration of clinical characteristics including age, response to different treatments, and recurrence rate in patients with different molecular subtypes, especially the hormone receptor-positive luminal vs. basal/triple-negative breast cancer (49–51). Second, the interaction between genes is a complex biological process; we only integrated existing differential-network-estimation methods to discover differential genes, which lacks innovation in methodology and comparability with other network-based approaches. Finally, the gene pre-screening process may omit some important characteristics. In the next step, we plan to focus on exploring the relationship between zinc-ion-related genes and breast cancer, and support our research through operational experiments.

## Conclusions

In conclusion, we constructed a breast cancer gene-interaction network and identified genes associated with long-term breast cancer survival. The results show that there is a strong correlation between the prognosis of breast cancer and zinc-ion binding. The screened genes can be used as new prognostic markers of breast cancer, providing a new development direction for clinical research and laying a foundation for subsequent research.

## Data Availability Statement

The original contributions presented in the study are included in the article/**Supplementary Material**. Further inquiries can be directed to the corresponding authors.

## Author Contributions

Conceptualization, CK and LL; Data curation, GY and YW; Formal analysis, YF, CK and FY; Funding acquisition, JJ and LL; Investigation, FY; Methodology, YH and JJ; Project ad-ministration, FW, YH, JJ and LL; Resources, GY and YW; Software, YF; Supervision, YH, JJ and LL; Validation, FY; Writing-original draft, YF and CK; Writing-review & editing, FW; Manuscript have been read and approved by all authors.

## Funding

This work was supported by General Program of China Postdoctoral Science Foundation (2021M691911), General Program of Natural Science Foundation of Shandong Province (ZR2021MH243), National Natural Science Foundation of China (81903410) and the Young Scholars Program of Shandong University.

## Conflict of Interest

The authors declare that the research was conducted in the absence of any commercial or financial relationships that could be construed as a potential conflict of interest.

## Publisher’s Note

All claims expressed in this article are solely those of the authors and do not necessarily represent those of their affiliated organizations, or those of the publisher, the editors and the reviewers. Any product that may be evaluated in this article, or claim that may be made by its manufacturer, is not guaranteed or endorsed by the publisher.
