# Supplementary material for: Integrated Multi-Omics Analysis Model to Identify Biomarkers Associated With Prognosis of Breast Cancer
Source: Front Oncol. 2022 Jun 10;12:899900. doi: 10.3389/fonc.2022.899900 (PMC9232398; doi:10.3389/fonc.2022.899900)
Supplement: Supplementary file 1 [file DataSheet_1.docx]

Supplementary Material for

Integrated multi-omics analysis model to identify biomarkers associated with prognosis of breast cancer

**Table S1.** The selected differential genes using DC-SIS method.

| Differential Genes | | | | | | |
| --- | --- | --- | --- | --- | --- | --- |
| ACAT1 | CD226 | EYA3 | MAML2 | PDCD1LG2 | RNF133 | TNFSF14 |
| ACER1 | CD3E | FSIP2 | MAP3K2 | PFDN2 | RNF208 | TNFSF9 |
| ACTR3 | CER1 | GALNT13 | MAT2B | PGAP1 | RPL31 | TRAF2 |
| AK3 | CHMP5 | GFI1 | MBP | PHAX | RPS25 | TRIM52 |
| AMOTL1 | CHST9 | GLB1L3 | MCTP1 | PINK1 | SCN3B | TRIM63 |
| ANGPTL5 | CNDP1 | GREB1L | MGAT4A | PLEKHB2 | SCN7A | TSGA10 |
| ANKRD24 | CXCR4 | HK3 | MGAT4B | PMPCA | SHD | TSPAN17 |
| ANKRD44 | DBN1 | HMMR | MIB1 | PNCK | SKA1 | UBE2E3 |
| ARL5A | DHRS9 | HSPA6 | MMP3 | PPP1R1C | SLC30A3 | UBXN11 |
| BCL10 | DOCK8 | HTR3A | MYOM1 | PRAMEF1 | SLCO6A1 | UNC50 |
| BCL9L | DOK3 | IFNA2 | N4BP3 | PROC | SMAD7 | USP28 |
| BNIP1 | DPAGT1 | IGSF8 | NDC80 | PRSS3 | SNCAIP | WDTC1 |
| BTNL9 | DPP10 | IL33 | NFATC1 | PSMG2 | SOX30 | YES1 |
| BUB1 | DSC1 | ISL1 | NLRX1 | PTPRS | SPDL1 | ZBTB37 |
| C11orf1 | DSEL | JAK2 | NME7 | PTTG1 | SPIRE1 | ZC3H6 |
| C11orf54 | ENOSF1 | KCNIP1 | NMI | R3HDM1 | SS18 | ZCCHC9 |
| CARF | EPB41 | KCNV2 | NRBP1 | RAB3GAP1 | SYTL1 | ZNF273 |
| CATSPER4 | EPHB2 | KCTD1 | OLA1 | RABL2A | TAF12 | ZNF354B |
| CCDC173 | EQTN | KHSRP | PADI4 | RLN1 | TMEFF2 | ZNF519 |
| CCNI2 | EVI5 | KMT2A | PDC | RND3 | TMEM163 | ZNF727 |

**Table S2.** The classification performance of mRNA expression data in TCGA.

| JDINAC | AUC | Sensitivity | Specificity | Accuracy |
| --- | --- | --- | --- | --- |
| 1-year | 0.716 (0.614, 0.818) | 0.879 | 0.514 | 0.865 |
| 3-year | 0.989 (0.984, 0.993) | 0.945 | 0.946 | 0.945 |
| 5-year | 0.813 (0.780, 0.847) | 0.713 | 0.781 | 0.729 |
| 10-year | 0.716 (0.614, 0.818) | 0.879 | 0.514 | 0.865 |
| LR | AUC | Sensitivity | Specificity | Accuracy |
| 1-year | 0.711 (0.661, 0.760) | 0.527 | 0.876 | 0.822 |
| 3-year | 0.710 (0.676, 0.743) | 0.663 | 0.686 | 0.672 |
| 5-year | 0.738 (0.700, 0.777) | 0.807 | 0.612 | 0.762 |
| 10-year | 0.743 (0.646, 0.839) | 0.937 | 0.600 | 0.924 |
| RF | AUC | Sensitivity | Specificity | Accuracy |
| 1-year | 0.879 (0.847, 0.911) | 0.733 | 0.844 | 0.827 |
| 3-year | 0.896 (0.877, 0.915) | 0.832 | 0.758 | 0.802 |
| 5-year | 0.881 (0.856, 0.907) | 0.858 | 0.714 | 0.825 |
| 10-year | 0.885 (0.816, 0.953) | 0.896 | 0.743 | 0.890 |

^*^LR and RF are the abbreviations for logistic regression and random forest respectively.

**Table S3.** The classification performance of mRNA expression data in METABRIC.

| JDINAC | AUC | Sensitivity | Specificity | Accuracy |
| --- | --- | --- | --- | --- |
| 1-year | 0.843 (0.771, 0.915) | 0.711 | 0.878 | 0.874 |
| 3-year | 0.841 (0.814, 0.868) | 0.719 | 0.811 | 0.798 |
| 5-year | 0.819 (0.796, 0.842) | 0.582 | 0.899 | 0.820 |
| 10-year | 0.865 (0.849, 0.881) | 0.685 | 0.867 | 0.772 |
| LR | AUC | Sensitivity | Specificity | Accuracy |
| 1-year | 0.721 (0.639, 0.803) | 0.489 | 0.986 | 0.974 |
| 3-year | 0.747 (0.714, 0.780) | 0.679 | 0.701 | 0.698 |
| 5-year | 0.709 (0.681, 0.736) | 0.627 | 0.713 | 0.691 |
| 10-year | 0.684 (0.660, 0.708) | 0.572 | 0.717 | 0.641 |
| RF | AUC | Sensitivity | Specificity | Accuracy |
| 1-year | 0.851 (0.789, 0.913) | 0.644 | 0.874 | 0.868 |
| 3-year | 0.878 (0.854, 0.903) | 0.727 | 0.847 | 0.831 |
| 5-year | 0.894 (0.878, 0.911) | 0.793 | 0.811 | 0.807 |
| 10-year | 0.902 (0.889, 0.915) | 0.820 | 0.778 | 0.800 |

^*^LR and RF are the abbreviations for logistic regression and random forest respectively.

**Table S4.** The enriched GO terms of the selected differential genes associated with long-term survival of breast cancer.

| **ID** | **GOTERM_BP_DIRECT** | **GOTERM_CC_DIRECT** | **GOTERM_MF_DIRECT** |
| --- | --- | --- | --- |
| **C11orf1** |  | GO:0005634~nucleus  GO:0005654~nucleoplasm |  |
| **IL33** | GO:0002282~microglial cell activation involved in immune response  GO:0002686~negative regulation of leukocyte migration  GO:0002826~negative regulation of T-helper 1 type immune response  GO:0002830~positive regulation of type 2 immune response  GO:0006351~transcription DNA-templated  GO:0032436~positive regulation of proteasomal ubiquitin-dependent protein catabolic process  GO:0032689~negative regulation of interferon-gamma production  GO:0032736~positive regulation of interleukin-13 production  GO:0032753~positive regulation of interleukin-4 production  GO:0032754~positive regulation of interleukin-5 production  GO:0032755~positive regulation of interleukin-6 production  GO:0042092~type 2 immune response  GO:0043032~positive regulation of macrophage activation  GO:0045944~positive regulation of transcription from RNA polymerase II promoter  GO:0050729~positive regulation of inflammatory response  GO:0051024~positive regulation of immunoglobulin secretion  GO:0051025~negative regulation of immunoglobulin secretion  GO:0051607~defense response to virus  GO:0061518~microglial cell proliferation  GO:0090197~positive regulation of chemokine secretion  GO:0097191~extrinsic apoptotic signaling pathway | GO:0005576~extracellular region  GO:0005615~extracellular space  GO:0005634~nucleus  GO:0005694~chromosome  GO:0016021~integral component of membrane  GO:0030133~transport vesicle | GO:0005125~cytokine activity  GO:0005515~protein binding |
| **OLA1** | GO:0046034~ATP metabolic process  GO:0098609~cell-cell adhesion | GO:0005730~nucleolus  GO:0005737~cytoplasm  GO:0005813~centrosome  GO:0005913~cell-cell adherens junction  GO:0016020~membrane  GO:0070062~extracellular exosome | GO:0005515~protein binding  GO:0005524~ATP binding  GO:0005525~GTP binding  GO:0016887~ATPase activity  GO:0043022~ribosome binding  GO:0043023~ribosomal large subunit binding  **GO:0046872~metal ion binding**  GO:0098641~cadherin binding involved in cell-cell adhesion |
| **RPL31** | GO:0000184~nuclear-transcribed mRNA catabolic process nonsense-mediated decay  GO:0002181~cytoplasmic translation  GO:0006364~rRNA processing  GO:0006412~translation  GO:0006413~translational initiation  GO:0006614~SRP-dependent cotranslational protein targeting to membrane  GO:0019083~viral transcription | GO:0005829~cytosol  GO:0005840~ribosome  GO:0005925~focal adhesion  GO:0016020~membrane  GO:0022625~cytosolic large ribosomal subunit  GO:0070062~extracellular exosome | GO:0003723~RNA binding  GO:0003735~structural constituent of ribosome  GO:0005515~protein binding  GO:0044822~poly(A) RNA binding |
| **SPDL1** | GO:0000132~establishment of mitotic spindle orientation  GO:0007062~sister chromatid cohesion  GO:0007080~mitotic metaphase plate congression  GO:0031577~spindle checkpoint  GO:0034501~protein localization to kinetochore  GO:0051301~cell division | GO:0000922~spindle pole  GO:0000940~condensed chromosome outer kinetochore  GO:0005634~nucleus  GO:0005815~microtubule organizing center  GO:0005829~cytosol | GO:0005515~protein binding  GO:0019899~enzyme binding  GO:0043515~kinetochore binding |
| **TMEM163** |  | GO:0030054~cell junction  GO:0030285~integral component of synaptic vesicle membrane  GO:0031901~early endosome membrane | **GO:0008270~zinc ion binding** |
| **TRAF2** | GO:0002726~positive regulation of T cell cytokine production  GO:0006461~protein complex assembly  GO:0006919~activation of cysteine-type endopeptidase activity involved in apoptotic process  GO:0007165~signal transduction  GO:0007249~I-kappaB kinase/NF-kappaB signaling  GO:0007250~activation of NF-kappaB-inducing kinase activity  GO:0010803~regulation of tumor necrosis factor-mediated signaling pathway  GO:0010939~regulation of necrotic cell death  GO:0030163~protein catabolic process  GO:0032743~positive regulation of interleukin-2 production  GO:0033209~tumor necrosis factor-mediated signaling pathway  GO:0034351~negative regulation of glial cell apoptotic process  GO:0034976~response to endoplasmic reticulum stress  GO:0042981~regulation of apoptotic process  GO:0043507~positive regulation of JUN kinase activity  GO:0043623~cellular protein complex assembly  GO:0046328~regulation of JNK cascade  GO:0050870~positive regulation of T cell activation  GO:0051023~regulation of immunoglobulin secretion  GO:0051091~positive regulation of sequence-specific DNA binding transcription factor activity  GO:0051092~positive regulation of NF-kappaB transcription factor activity  GO:0051291~protein heterooligomerization  GO:0051865~protein autoubiquitination  GO:0070059~intrinsic apoptotic signaling pathway in response to endoplasmic reticulum stress  GO:0070207~protein homotrimerization  GO:0070534~protein K63-linked ubiquitination  GO:0071550~death-inducing signaling complex assembly  GO:0071732~cellular response to nitric oxide  GO:0090073~positive regulation of protein homodimerization activity  GO:0097296~activation of cysteine-type endopeptidase activity involved in apoptotic signaling pathway  GO:0097300~programmed necrotic cell death  GO:1901215~negative regulation of neuron death  GO:1902041~regulation of extrinsic apoptotic signaling pathway via death domain receptors  GO:1902042~negative regulation of extrinsic apoptotic signaling pathway via death domain receptors  GO:1903265~positive regulation of tumor necrosis factor-mediated signaling pathway  GO:1903721~positive regulation of I-kappaB phosphorylation  GO:2001238~positive regulation of extrinsic apoptotic signaling pathway | GO:0000151~ubiquitin ligase complex  GO:0005622~intracellular  GO:0005737~cytoplasm  GO:0005829~cytosol  GO:0005938~cell cortex  GO:0009898~cytoplasmic side of plasma membrane  GO:0012506~vesicle membrane  GO:0035631~CD40 receptor complex  GO:0045121~membrane raft  GO:0097057~TRAF2-GSTP1 complex  GO:1990597~AIP1-IRE1 complex  GO:1990604~IRE1-TRAF2-ASK1 complex | GO:0004842~ubiquitin-protein transferase activity  GO:0004871~signal transducer activity  GO:0005164~tumor necrosis factor receptor binding  GO:0005174~CD40 receptor binding  GO:0005515~protein binding  **GO:0008270~zinc ion binding**  GO:0016874~ligase activity  GO:0019899~enzyme binding  GO:0019901~protein kinase binding  GO:0019903~protein phosphatase binding  GO:0031435~mitogen-activated protein kinase kinase kinase binding  GO:0031625~ubiquitin protein ligase binding  GO:0031996~thioesterase binding  GO:0032403~protein complex binding  GO:0042802~identical protein binding  GO:0046625~sphingolipid binding |
| **TRIM52** | GO:0051092~positive regulation of NF-kappaB transcription factor activity | GO:0005622~intracellular | **GO:0008270~zinc ion binding** |
| **TSGA10** | GO:0007283~spermatogenesis  GO:0030031~cell projection assembly | GO:0005737~cytoplasm  GO:0031514~motile cilium  GO:0031965~nuclear membrane  GO:0043005~neuron projection | GO:0005515~protein binding |
| **TSPAN17** | GO:0007166~cell surface receptor signaling pathway  GO:0016567~protein ubiquitination  GO:0045747~positive regulation of Notch signaling pathway  GO:0072594~establishment of protein localization to organelle  GO:0090002~establishment of protein localization to plasma membrane | GO:0000151~ubiquitin ligase complex  GO:0005887~integral component of plasma membrane  GO:0016021~integral component of membrane | GO:0004842~ubiquitin-protein transferase activity  GO:0019899~enzyme binding |
| **USP28** | GO:0000077~DNA damage checkpoint  GO:0006281~DNA repair  GO:0006511~ubiquitin-dependent protein catabolic process  GO:0006974~cellular response to DNA damage stimulus  GO:0007265~Ras protein signal transduction  GO:0008283~cell proliferation  GO:0010212~response to ionizing radiation  GO:0016579~protein deubiquitination  GO:0031647~regulation of protein stability  GO:0034644~cellular response to UV  GO:0042771~intrinsic apoptotic signaling pathway in response to DNA damage by p53 class mediator | GO:0005634~nucleus  GO:0005654~nucleoplasm  GO:0005730~nucleolus  GO:0005737~cytoplasm  GO:0043234~protein complex | GO:0004843~thiol-dependent ubiquitin-specific protease activity  GO:0005515~protein binding  GO:0036459~thiol-dependent ubiquitinyl hydrolase activity |
| **ZBTB37** | GO:0006351~transcription DNA-templatedGO:0006355~regulation of transcription DNA-templated | GO:0005634~nucleus | GO:0003676~nucleic acid binding  GO:0003677~DNA binding  **GO:0046872~metal ion binding** |
| **ZNF273** | GO:0006351~transcription DNA-templatedGO:0006355~regulation of transcription DNA-templated | GO:0005622~intracellular  GO:0005634~nucleus | GO:0000978~RNA polymerase II core promoter proximal region sequence-specific DNA binding  GO:0003676~nucleic acid binding  GO:0005515~protein binding  **GO:0046872~metal ion binding** |
| **ZNF519** | GO:0006351~transcription DNA-templated  GO:0006355~regulation of transcription DNA-templated  GO:0007283~spermatogenesis  GO:0051038~negative regulation of transcription involved in meiotic cell cycle | GO:0005622~intracellular  GO:0005634~nucleus | GO:0000978~RNA polymerase II core promoter proximal region sequence-specific DNA binding  GO:0003676~nucleic acid binding  **GO:0046872~metal ion binding** |
| **ZNF727** | GO:0006351~transcription DNA-templated  GO:0006355~regulation of transcription DNA-templated | GO:0005634~nucleus | GO:0000978~RNA polymerase II core promoter proximal region sequence-specific DNA binding  **GO:0046872~metal ion binding** |


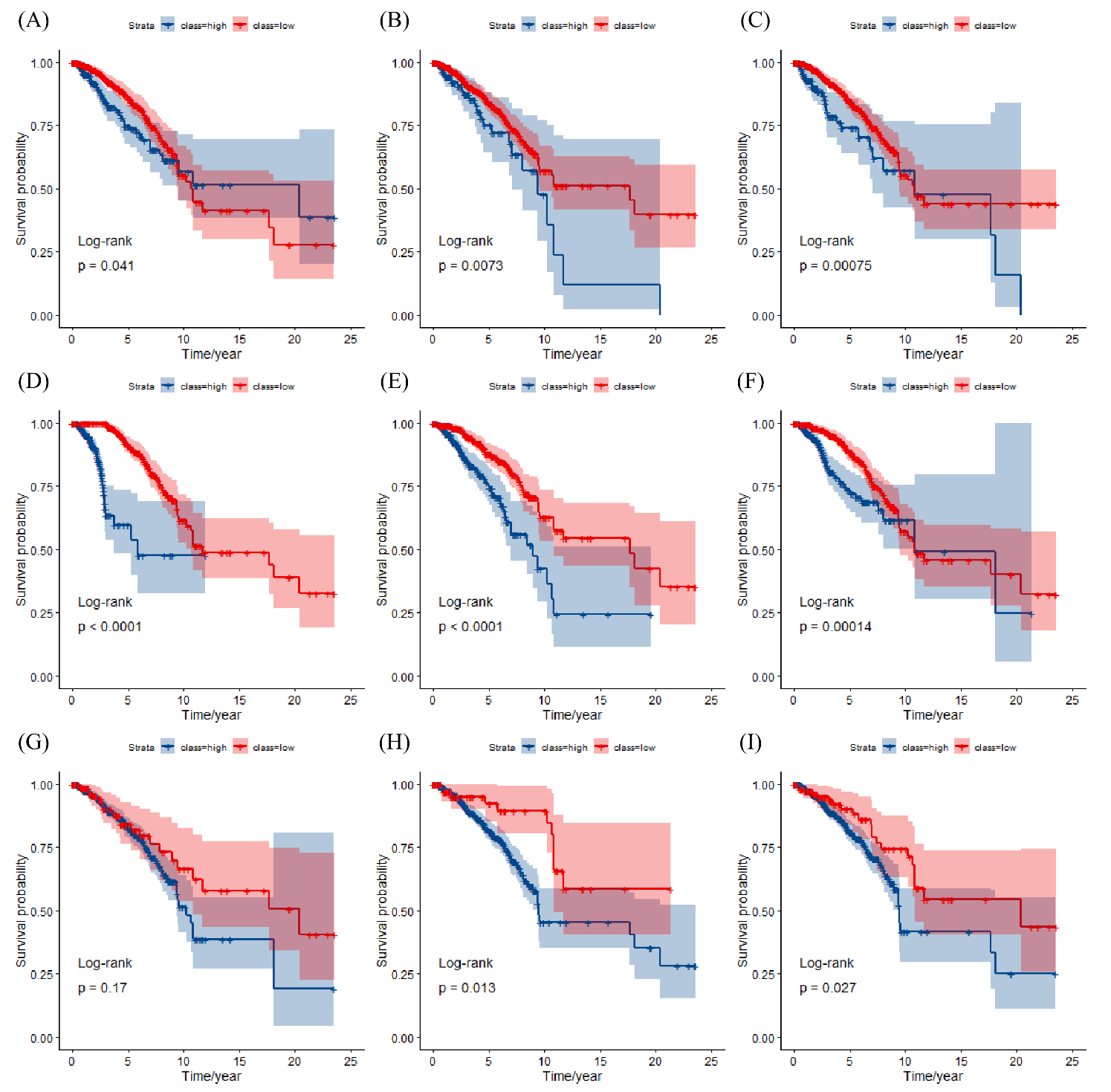


**Figure S1. Kaplan-Meier curves for overall survival of TCGA** based on 1-year classifiers **(A)** JDINAC, **(B)** Logistic regression, and **(C)** Random forest; 3-year classifiers **(D)** JDINAC, **(E)** Logistic regression, and **(F)** Random forest; and 10-year classifiers **(G)** JDINAC, **(H)** Logistic regression, and **(I)** Random forest.


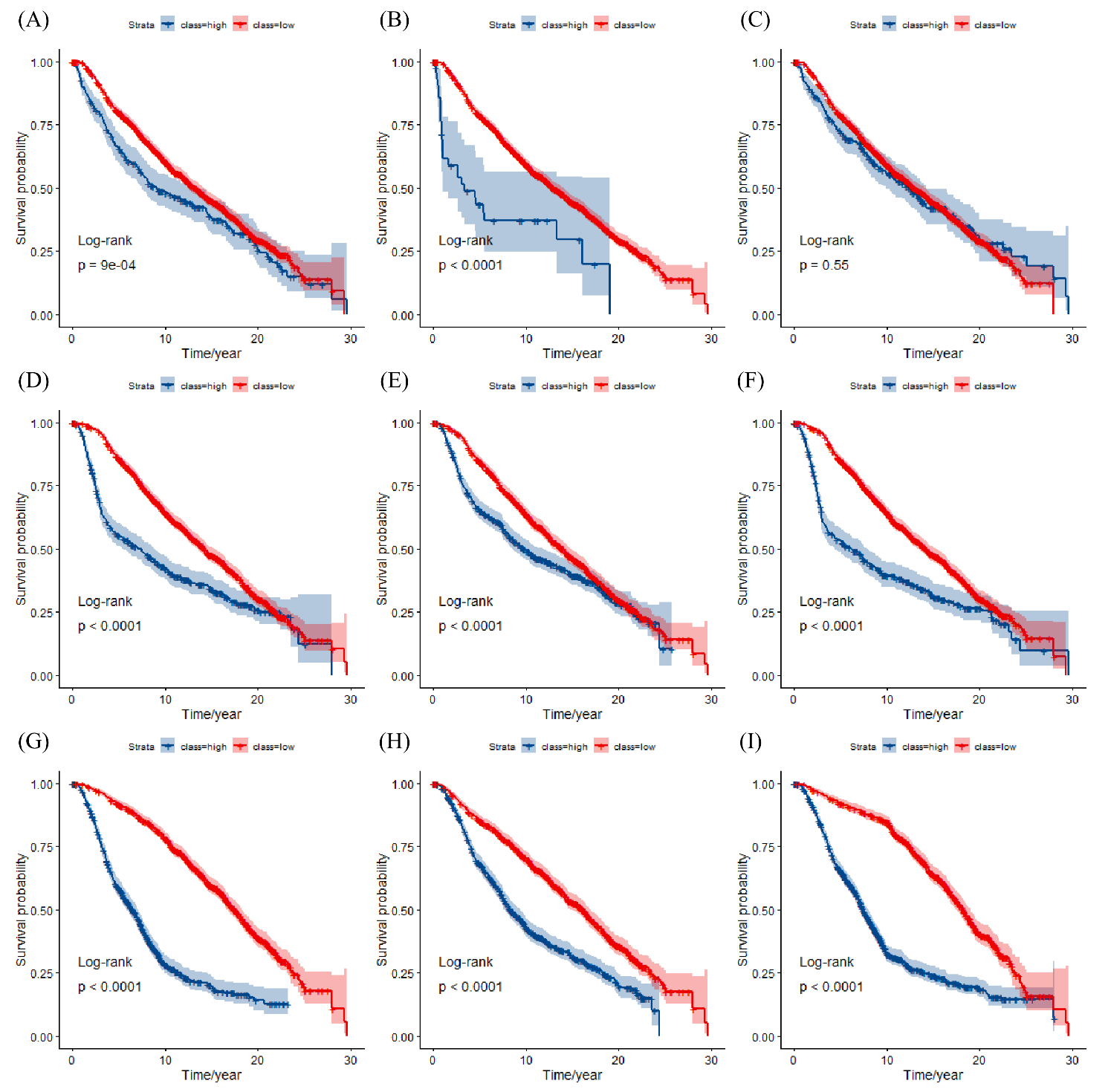


**Figure S2.** **Kaplan-Meier curves for overall survival of METABRIC** based on 1-year classifiers **(A)** JDINAC, **(B)** Logistic regression, and **(C)** Random forest; 3-year classifiers **(D)** JDINAC, **(E)** Logistic regression, and **(F)** Random forest; and 10-year classifiers **(G)** JDINAC, **(H)** Logistic regression, and **(I)** Random forest.
